# The Effects of Microencapsulation Technology on the Flavor Quality of *Zanthoxylum* Oil Based on E-Nose, GC–IMS, and GC–MS

**DOI:** 10.3390/molecules30163366

**Published:** 2025-08-13

**Authors:** Liangyun Wang, Jia Chen, Xuemei Cai, Dandan Li, Xinxin Zhao, Yu Fu, Lei Huang, Yi Rao, Yuwen Yi, Mingfeng Qiao, Baohe Miao

**Affiliations:** 1 Culinary Science Key Laboratory of Sichuan Province, Sichuan Tourism University, Chengdu 610100, China; 0000409@sctu.edu.cn (L.W.); 17345221518@163.com (J.C.); cxm121517@163.com (X.C.); 18200469476@163.com (D.L.); jeff-800911@126.com (Y.Y.); 2 College of Food Science and Technology, Southwest Minzu University, Chengdu 610041, China; yufu@swu.edu.cn; 3 Institute of Urban Agriculture, Chinese Academy of Agricultural Sciences, Chengdu National Agricultural Science & Technology Center, Chengdu 610213, China; 15008313421@163.com; 4 Chengdu Railway Chuanzhiwei Travel Service Co., Ltd., Chengdu 610084, China; 9630674@163.com (L.H.); 15982103129@163.com (Y.R.)

**Keywords:** *Zanthoxylum* oil, microencapsulation technology, GC–MS, GC–IMS, flavor quality

## Abstract

To investigate the impact of microencapsulation on the volatile organic compounds (VOCs) in *Zanthoxylum* oil, this study compared unencapsulated Zanthoxylum oil (ZO) with microencapsulated Zanthoxylum oil (MZO) using physicochemical analysis, sensory evaluation, and molecular sensory analysis. Sensory evaluation revealed significant differences in aroma attributes between ZO and MZO, whereas no notable differences were observed in numbing intensity or overall acceptability. Colorimetric analysis indicated significant distinctions between the two samples. Electronic nose (E-nose) analysis demonstrated a reduction in overall aroma intensity for MZO compared to ZO. Gas chromatography–mass spectrometry (GC–MS) identified 43 VOCs, including 22 compounds present in both samples, accounting for 46.8% of the total. Terpenes represented the predominant class in both ZO (69.7%) and MZO (68.2%). Comprehensive analysis based on odor activity value (OAV) and variable importance in projection (VIP) identified nine volatile compounds as key aroma contributors. Gas chromatography–ion mobility spectrometry (GC–IMS) detected 90 the volatile organic compounds (VOCs), with esters (30.38%) and heterocyclic compounds (10.42%) predominating in ZO, while esters (29.08%) and alcohols (26.12%) were predominant in MZO. Compared to ZO, MZO exhibited increased levels of alcohols (from 12.04% to 26.12%) and terpenes (from 1.39% to 3.53%), but decreased levels of acids (from 5.77% to 2.72%) and aldehydes (from 10.29% to 4.62%). This approach provides a comprehensive assessment of flavor quality before and after microencapsulation, offers a scientific basis for quality control, and facilitates the development and utilization of Zanthoxylum oil resources.

## 1. Introduction

*Zanthoxylum bungeanum*, a member of the genus Zanthoxylum in the Rutaceae family, is widely distributed throughout Asian countries, including China, India, and South Korea. As both a traditional Chinese spice and a medicinal herb, it possesses significant economic value owing to its dual applications in culinary and medicinal contexts [1]. Studies have shown that *Zanthoxylum bungeanum* contains a rich array of bioactive components, including volatile oils, alkaloids, amides, flavonoids, and polyphenolic compounds [2,3]. In China, *Zanthoxylum bungeanum* occupies a prominent position as a traditional spice in food flavoring, primarily due to its distinctive numbing sensation and unique aroma. The characteristic numbing effect is mainly attributed to amide compounds, including α-sanshool and hydroxy-α-sanshool, while its aroma is largely derived from monoterpenes and sesquiterpenes present in the volatile oils, such as linalool, limonene, and β-caryophyllene.

Nowadays, *Zanthoxylum bungeanum* is mainly processed and utilized through deep and comprehensive processing technologies in forms such as whole peppercorns, *Zanthoxylum bungeanum* powder, and *Zanthoxylum* oil [4]. However, these forms fail to fully disrupt the cellular structure of *Zanthoxylum bungeanum*, resulting in incomplete release of flavor compounds and poor product stability [5]. During storage, *Zanthoxylum bungeanum* is also susceptible to factors such as temperature, oxygen, light, and moisture, which can alter its aroma, color, and flavor, ultimately affecting product quality and market value [6]. Microencapsulation technology can effectively microencapsulated *Zanthoxylum* oil within semi-permeable or sealed capsule membranes, mitigating the impact of external factors on the oil. Microencapsulation technology effectively encapsulates *Zanthoxylum* oil within semi-permeable or sealed capsule membranes, mitigating degradation caused by external factors. This technology is critical for preserving *Zanthoxylum* oil, whose unique composition—including heat-sensitive volatile terpenes and bioactive sanshools (responsible for its characteristic numbing sensation)—makes it highly susceptible to degradation during processing. For example, spray drying, during which temperatures exceed 150 °C and prolonged oxygen exposure occurs, can induce a 30–40% loss of volatile compounds and oxidative degradation of sanshools [7,8]. Yuan et al. [9] prepared algal oil microcapsules with excellent storage stability by employing complex coacervation using soybean protein and chitosan as composite wall materials. Meng et al. [10] selected polysaccharides and proteins as composite wall materials to microencapsulate *Zanthoxylum* oil, effectively protecting the numbing flavor compounds from degradation. Nevertheless, the intricate flavor profile of *Zanthoxylum* oil presents significant challenges for traditional analytical methods to fully capture its complexity, as few systematic investigations have addressed the impact of microencapsulation technology on its flavor quality.

At present, the rapid development of advanced analytical techniques, such as electronic noses (E-nose), gas chromatography–mass spectrometry (GC–MS), and gas chromatography–ion mobility spectrometry (GC–IMS), has provided powerful tools for investigating food flavor profiles. Feng et al. [11] used headspace solid-phase microextraction gas chromatography–mass spectrometry (HS-SPME-GC–MS) and headspace gas chromatography–ion mobility spectrometry (HS-GC–IMS) techniques to determine the volatile organic compounds (VOCs) in eight different Chinese geographical indication red and green *Zanthoxylum* species. Gao et al. [12] examined the aroma sensory characteristics and VOCs of seven green Zanthoxylum samples from the Sichuan–Chongqing region using GC–MS in combination with odor activity value (OAV) analysis. Niu et al. [13] employed HS-SPME-GC–MS to qualitatively and quantitatively analyze the volatile flavor components in *Zanthoxylum* oil. The E-nose employs a sensor array that simulates the human olfactory system, enabling rapid discrimination of complex odors and providing fast and accurate detection during *Zanthoxylum* oil production. GC–MS is employed for the precise identification and quantitative analysis of flavor components, thereby elucidating the specific influence of microencapsulation technology on pivotal flavor compounds [14]. Meanwhile, GC–IMS, with its high sensitivity, facilitates the intuitive evaluation of the protective effects of microencapsulation technology on flavor compounds [15,16]. The integration of these techniques is vital for elucidating the effects of microencapsulation on Z*anthoxylum* oil flavor, enhancing product quality, and comprehensively evaluating its flavor profile.

This study focuses on microencapsulated *Zanthoxylum* oil by integrating advanced sensory instruments, including GC–MS, GC–IMS, and E-nose, together with sensory evaluation and physicochemical analysis. The research compares the volatile components between *Zanthoxylum* oil (ZO) and microencapsulated *Zanthoxylum* oil (MZO), explores the influencing factors and mechanisms of flavor formation, screens and analyzes major aroma compounds and characteristic differential substances, and establishes a fingerprint profile of characteristic flavors. The results provide both theoretical and practical foundations for the quality evaluation and classification of microencapsulated *Zanthoxylum* oil, offer significant guidance for quality control, and promote the development and utilization of *Zanthoxylum* oil resources.

## 2. Results and Discussion

### 2.1. Physicochemical Properties Analysis

#### 2.1.1. Sensory Analysis

**Figure 1:**
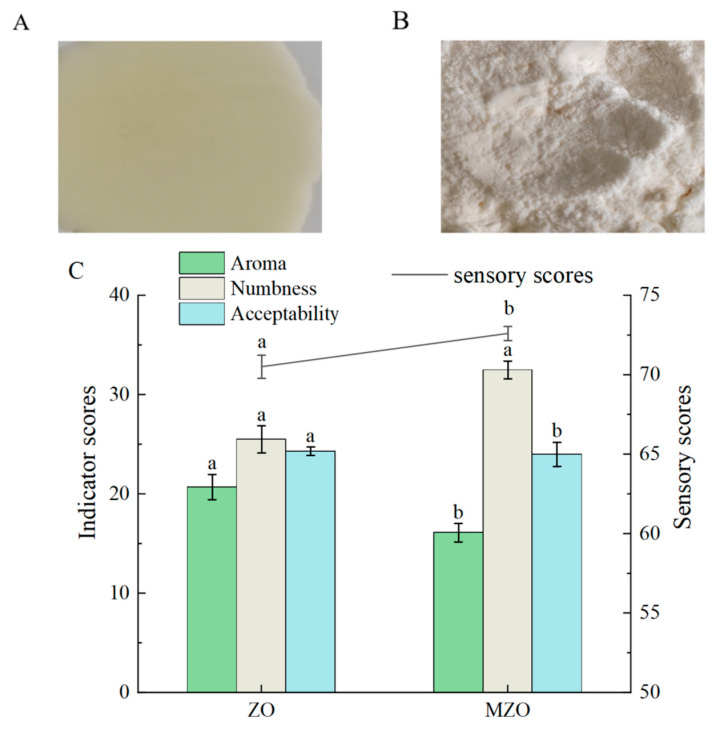
Sensory profiling of ZO and MZO. (**A**) Macroscopic morphology figures of ZO, (**B**) Macroscopic morphology figures of MZO, (**C**) Sensory scores. The different letters show a significant difference between samples in scores according to Duncan’s multiple range test (*p* < 0.05).

Sensory evaluation is the most direct, rapid, and efficient method for assessing food quality using human sensory organs, including vision, taste, smell, and touch. Figure 1A,B display the macroscopic morphology of two distinct *Zanthoxylum* oil samples. Significant differences in physical state and coloration can be observed between the two formulations. Figure 1A represents ZO, presenting as a pale yellow oily liquid with intense aromatic and numbing notes detectable olfactorily. In contrast, Figure 1B illustrates MZO, characterized by an off-white powdered form that demonstrates subdued numbing aroma and fragrance olfactorily; however, a pronounced numbing sensation emerges upon oral evaluation, aligning with the sensory evaluation outcomes. Figure 1C presents a histogram based on the sensory evaluation results of *Zanthoxylum* oil and its microencapsulated form. The histogram reveals a significant difference in the aroma dimension between the two forms of the oil, with the microencapsulated *Zanthoxylum* oil scoring higher in the numbing taste dimension. However, the difference in overall acceptability is not significant. This indicates that the microencapsulation process has altered the scent of the *Zanthoxylum* oil while effectively preserving its numbing taste. The observed changes in aroma may be attributed to the loss of certain volatile compounds due to the high temperatures involved in spray drying or the generation of unpleasant odors. Further research is required to determine the precise causes of these changes.

#### 2.1.2. Analysis of Colorimetric

The color difference of the samples was analyzed using a colorimeter, and the chroma value (C*), hue value (h*), and color difference value (Δ*E*) were calculated. The results of the color difference analysis were presented in Table 1. As shown in Table 1, significant differences (*p* < 0.05) were observed in the L*, a*, b*, C*, and Δ*E* values of the samples. Specifically, MZO exhibited higher L*, a*, b*, and C* values compared to ZO, indicating significant differences in brightness, red–green intensity, and yellow–blue intensity. The Δ*E* value of the MZO was 10.13, while ZO was 20.12, demonstrating a substantial difference and confirming a significant distinction between the two sample groups [17].

#### 2.1.3. E-Nose Analysis

**Figure 2:**
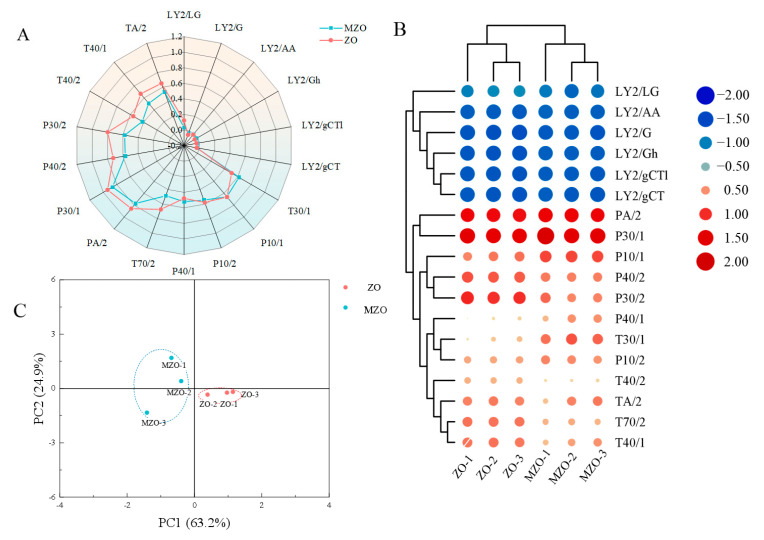
E-nose analysis of ZO and MZO. (**A**) The radar chart of the E-nose, (**B**) E-Nose clustering heatmap, (**C**) principal component analysis of the E-nose.

E-nose is an intelligent sensory system designed to mimic human olfaction, characterized by high sensitivity, rapid detection, and robust reproducibility. When integrated with chemometric data models, it enables the visualization of analytical results [18]. In this study, E-nose analysis was performed on both native ZO and MZO. Radar plots were constructed using sensor response values recorded at 120 s (Figure 2A). Significant differences (*p* < 0.05) were observed between ZO and MZO in sensors TA/2, T40/1, T40/2, P30/2, P40/2, T70/2, and T30/1, indicating a marked reduction in odor intensity and partial loss of volatile components in MZO after microencapsulation.

For sensors PA/2 and P30/1, both ZO and MZO exhibited response values ranging from 0.8 to 1.0. However, ZO displayed higher responses (0.6–0.8) than MZO (lower range) in sensors T70/2, TA/2, T40/1, P30/2, and P40/2, consistent with the hypothesis that aroma intensity correlates positively with sensor response magnitudes. Notably, the LY2 sensor showed overlapping response values (−0.2–0.2) for both samples, suggesting negligible differences in aromatic composition for this specific sensor. Overall, the higher response values of ZO compared to MZO align with chromatographic fingerprinting data, confirming that microencapsulation effectively mitigates volatile component loss. Cluster heatmap analysis of electronic nose data successfully discriminated between ZO and MZO, with key sensors (S2, S7, S9) contributing to the separation. Samples LY2/G, LY2/Gh, LY2/LG, LY2/AA, LY2/gCT, and LY2/gCTI formed a distinct cluster, indicating similar aroma profiles, while PA/2, P30/1, P40/2, P10/1, and P30/2 exhibited unique sensor response patterns (Figure 2B). Based on the data shown in Figure 2C, it can be concluded that the first and second principal components account for over 85% of the PCA analysis [19]. This indicates that the main flavor profile of the sample was accurately represented and suggests that the samples are reliable and consistent. By combining the radar chart’s signal intensity distribution with the PCA results, it was apparent that MZO closely resemble the original flavor characteristics of ZO.

While the E-nose efficiently discriminates global odor profiles, it lacks specificity for identifying individual odor-active compounds. To elucidate molecular-level changes, complementary GC–MS analysis is required to characterize the specific volatiles altered during microencapsulation.

### 2.2. GC–MS Analysis

#### 2.2.1. Analysis of VOCs Composition by GC–MS

**Figure 3:**
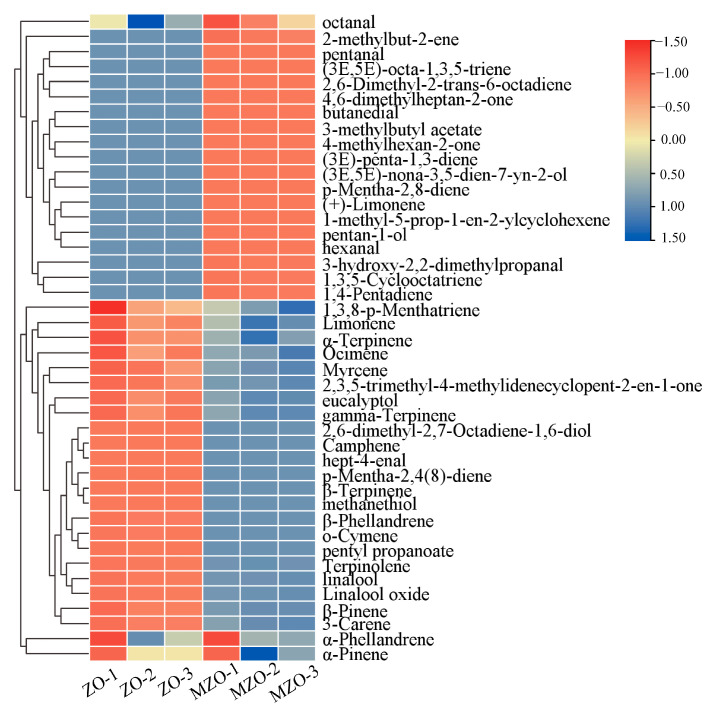
Heatmap clustering of the content of different types of VOCs before and after microencapsulation treatment by GC–MS.

To further clarify the changes in specific aroma compounds of *Zanthoxylum* oil after microencapsulation, the experiment analyzed both ZO and microencapsulated MZO using GC–MS. The analytical results are shown in Supplementary Table S1. A total of 43 compounds were detected in ZO and MZO, including 8 hydrocarbons, 17 terpenes, 6 alcohols, 6 aldehydes, 2 esters, and 4 ketones. Compounds are grouped by chemical class. terpenes and alcohols were the main volatile components in both ZO and MZO. There were 22 shared compounds: β-phellandrene, α-phellandrene, α-pinene, gamma-terpinene, myrcene, terpinolene, limonene, ocimene, 1,3,8-*p*-menthatriene, α-terpinene, β-pinene, 3-carene, *p*-mentha-2,4(8)-diene, 1,4-pentadiene, eucalyptol, *o*-cymene, β-terpinene, linalool, linalool oxide, octanal, pentyl propanoate, and 2,3,5-trimethyl-4-methylidenecyclopent-2-en-1-one. Among these shared compounds, 3 were hydrocarbons, 14 were terpenes, 2 were alcohols, and 1 each was an aldehyde, ester, and a ketone. Figure 3 presents a cluster heatmap, clearly demonstrating the changes in concentration of multiple VOCs before and after microencapsulation of *Zanthoxylum* oil.

Terpenes were the primary volatile components in *Zanthoxylum*, mainly derived from the oxidative breakdown of alkyl chains in fatty acids within the spice, leading to the formation of free radicals. The relative contents of terpenes in ZO and MZO were 69.7% and 68.2%, respectively, making them the highest among all volatile compounds. A total of 14 terpenes compounds were shared between the two samples, with those having contents greater than 1% including phellandrene, gamma-terpinene, myrcene, limonene, ocimene, *p*-menthatriene, terpinene, and β-pinene, which are the main volatile components. The contents of these shared compounds were generally high, indicating that terpenes were not significantly affected by spray drying. Gao et al. [20] used GC–MS to analyze the main volatile components of red *Zanthoxylum* oil and found that terpenes were the most abundant, with myrcene, limonene, ocimene, sabinene, and β-pinene being the primary volatile substances [21]. These findings were largely consistent with the results of this study. Among the main shared compounds, the contents in MZO were lower than those in ZO, which may be attributed to losses during the microencapsulation process (due to the high temperature of spray drying). It was reported that the terpenes components in *Zanthoxylum* oleoresin and microencapsulated *Zanthoxylum* oil were primarily β-phellandrene, myrcene, limonene, carene, gamma-terpinene, and caryophyllene, which aligns well with the conclusions of this study. 2-Methyl-2-butene, a colorless volatile compound with an unpleasant odor, was detected in MZO at a content exceeding 10%, but it was not detected in *Zanthoxylum* oil. The presence of 2-methyl-2-butene is likely associated with the microencapsulation process or additives, possibly arising from thermal cracking during spray drying. The high-temperature environment (inlet 180 °C, outlet 80 °C) may cleave C-C bonds in parent terpenoids, generating smaller alkenes via radical-mediated fragmentation, and further research is needed to determine the specific cause.

Studies have shown that the formation of alcohol compounds primarily originates from the oxidation of polyunsaturated fatty acids (PUFAs). Specifically, when these fatty acids undergo oxidation, they produce hydroperoxides, which further decompose to form alcohol compounds [22]. Among these alcohols, while straight-chain saturated alcohols contribute limitedly to the overall aroma, their aromatic intensity increases with the elongation of the carbon chain, imparting diverse flavor profiles such as fatty, fresh, and woody notes. In contrast, unsaturated alcohols play a more critical role in the overall flavor composition due to their lower flavor thresholds [23]. A total of 6 alcohol compounds were detected, with three shared compounds, namely, linalool, linalool oxide, and eucalyptol, all of which had relative contents exceeding 1% and were identified as the primary alcohol-based volatile components. These three compounds exhibited a decreasing trend in MZO, likely due to the high-temperature spray drying process. It was reported that the alcohol compounds in *Zanthoxylum* oleoresin and microencapsulated *Zanthoxylum* oil included linalool, α-terpineol, and nerolidol, with linalool being the predominant alcohol-based volatile substance [24]. These findings are largely consistent with the results of this study.

Furthermore, octanal was identified as the most abundant shared compound among aldehydes. In summary, the contents of hydrocarbons and aldehydes in MZO were lower than those in ZO. This observation may explain why, in E-nose detection, the response intensity of MZO was generally lower on most sensors compared to that of ZO.

#### 2.2.2. Analysis of Odor Activity Values

To elucidate the active odor-contributing ingredients and understand their contributions, an odor activity value (OAV) analysis was conducted on the GC–MS results. Subsequently, the VOC data obtained underwent thorough analysis, and the results are detailed in Supplementary Table S1. A total of 22 compounds with OAV ≥ 1 were detected, with 16 compounds identified in both ZO and MZO. The shared compounds with 100 > OAV ≥ 1 included linalool oxide, α-phellandrene, β-phellandrene, α-pinene, gamma-terpinene, terpinolene, α-terpinene, β-pinene, and 3-carene, totaling nine compounds. The shared compounds with OAV ≥ 100 were linalool, eucalyptol, octanal, myrcene, limonene, and 1,3,8-*p*-menthatriene, totaling six compounds. The presence of these compounds indicates that the primary volatile components of ZO were not lost during the spray drying process for microencapsulation, and the main aroma profile remained unchanged. The content of linalool in MZO was markedly reduced (from 6.17% to 1.44%). However, due to its remarkably low aroma threshold, all OAV values remained above 6000. Linalool, a monoterpene alcohol with flowery and citrusy aromas, has been confirmed to possess pharmacological effects such as anti-inflammatory, sedative, and sleep-promoting properties [25,26]. This compound has an extremely low aroma threshold in water (0.00022 mg/kg), indicating that even at very low concentrations, linalool can significantly contribute to the aroma profile. Studies have shown that linalool is synthesized through the catalysis of linalool synthase, which converts geranyl diphosphate into linalool [27]. Notably, hept-4-enal (OAV = 4.00) and pentyl propanoate (OAV = 6.83) were exclusively or predominantly detected in ZO, while pentanal (OAV = 3.33), hexanal (OAV = 4.00), 3-methylbutyl acetate (OAV = 66.67), and 4-methylhexan-2-one (OAV = 12.35) were either newly detected or showed substantially higher odor activity values in MZO, suggesting these compounds may serve as characteristic flavor substances differentiating the two samples.

Octanal, a primary volatile compound in the samples, exhibited a content exceeding 4% and an aroma threshold of 0.00059 mg/kg, resulting in OAV values surpassing 8000. It imparts a grassy note and has been demonstrated to originate from the oxidative breakdown of n-9 polyunsaturated fatty acids containing oleic acid. Myrcene, a terpenoid compound with significant bioactivity and broad applications, was present in the samples at levels above 8% [28]. Widely utilized in the fragrance industry as a key raw material for synthesizing colognes and deodorants, myrcene is also notable for its pharmacological properties, including analgesic, antioxidant, and anti-inflammatory effects [29]. Due to its low aroma threshold, its OAV in the samples exceeded 6000, indicating that even trace amounts substantially influence the overall aroma profile. As a critical odor-active substance in spices, myrcene is not only derived from natural sources but can also be obtained through thermal cracking of β-pinene or via the synthesis of linalool. Eucalyptol, a major volatile compound in the samples, was detected at levels exceeding 2%, with an aroma threshold of 0.00110 mg/kg and OAV values surpassing 2000. It exhibits camphoraceous and cooling herbal notes and has been demonstrated to possess pharmacological properties such as anti-inflammatory, antioxidant, antibacterial, bronchodilatory, and analgesic effects. Studies indicate that eucalyptol can be generated via intramolecular hydroxyl-ene cyclization of α-terpineol or through autocyclization of 4-terpineol [30,31].

#### 2.2.3. Analysis of Multivariate Statistical

**Figure 4:**
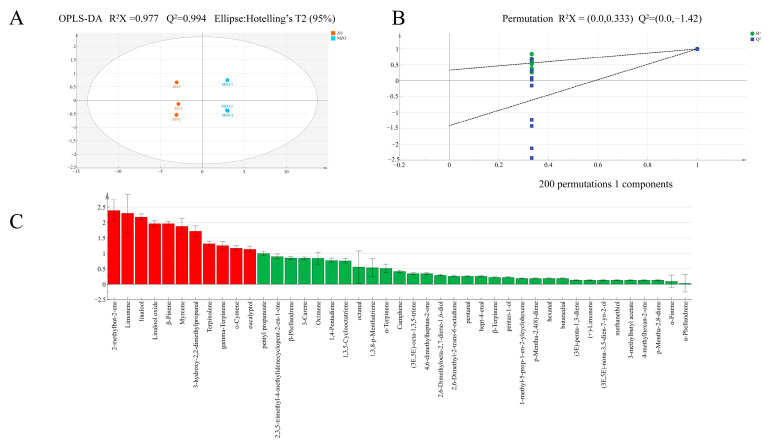
OPLS-DA model of VOCs before and after microencapsulation treatment. (**A**) Score plot; (**B**) cross-validation; (**C**) VIP scores. Red sections represent chemical substances with VIP > 1, and green sections represent chemical substances with VIP < 1.

To delve deeper into the flavor distinctions among ZO and MZO, we employed OPLS-DA, an enhancement of PLS-DA incorporating orthogonal transformation corrections, segregates the X matrix into two distinct categories of information: those related and unrelated to Y. This enables the screening of differential variables, thereby facilitating the elimination of those that are unrelated. This model’s cumulative statistical value R^2^X stands at 0.977, the model explanation rate parameter R^2^Y at 0.996, and the predictive ability parameter Q^2^ at 0.994 (Figure 4A). All exceed the 0.5 threshold, testifying to the model’s robust explanatory power in analyzing flavor differences in ZO and MZO. This result indicates that the ZO and MZO samples had significant differences in overall odor, possibly because of the influence of microencapsulation on the flavor of *Zanthoxylum* oil, corroborating the findings of the E-nose analysis. To further verify the reliability of the model, a 200-times cross-validation was conducted, which revealed an R^2^ slope greater than zero and a Q^2^ intercept less than zero, indicating that the model was not overfitted and could be analyzed further (Figure 4B). VIP (variable importance in projection) values, illustrated in Figure 4C, identified 11 volatile compounds with VIP values surpassing 1. A comparison of the key variable compounds with OAV > 1 and VIP > 1 pinpointed nine volatile compounds as characteristic substances: limonene, linalool, linalool oxide, β-pinene, myrcene, terpinolene, gamma-terpinene, *o*-cymene, and eucalyptol.

### 2.3. Correlation Analysis Between E-Nose and GC–MS

**Figure 5:**
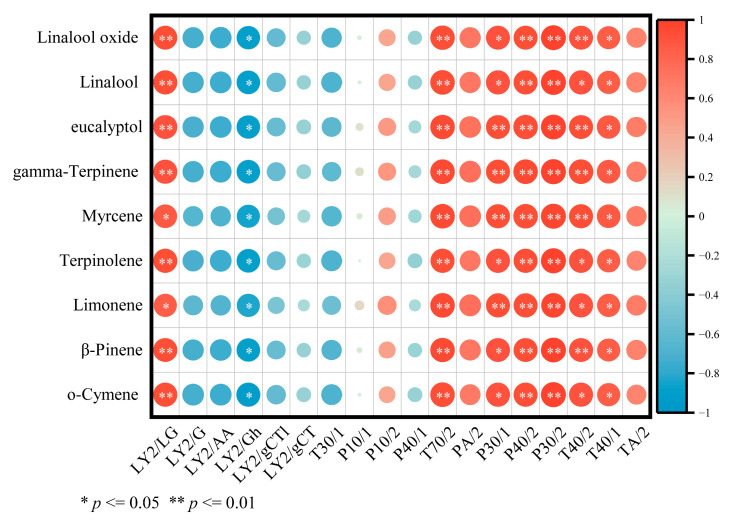
The Pearson’s correlation heatmap illustrates the correlation between the significantly altered levels of volatile compounds and the E-nose sensor responses. In the heatmap, colors represent correlation coefficients, with red indicating positive correlations and blue indicating negative correlations. Here, ** indicates extremely significant differences (*p* < 0.01), while * denotes significant differences (*p* < 0.05).

To improve the overall effectiveness of both E-nose and GC–MS, an investigation was conducted to explore the potential correlation between E-nose sensor responses and volatile compound levels detected using GC–MS. As depicted in Figure 5, several sensors including LY2/LG, T70/2, P30/1, P30/2, P40/2, T40/2, and T40/1 showed a positive correlation with key aroma components, which were identified through analysis of OAV and OPLS-DA. Conversely, sensors, LY2/G, LY2/AA, LY2/Gh, and T30/1 exhibited a negative correlation with key aroma components. These results suggested that the E-nose is a reliable tool for assessing the flavor profile of *Zanthoxylum* oil with and without microencapsulation based on the correlation between key aroma components and E-nose sensor response values.

### 2.4. GC–IMS Analysis

#### 2.4.1. GC–IMS 2D Mapping Analysis

**Figure 6:**
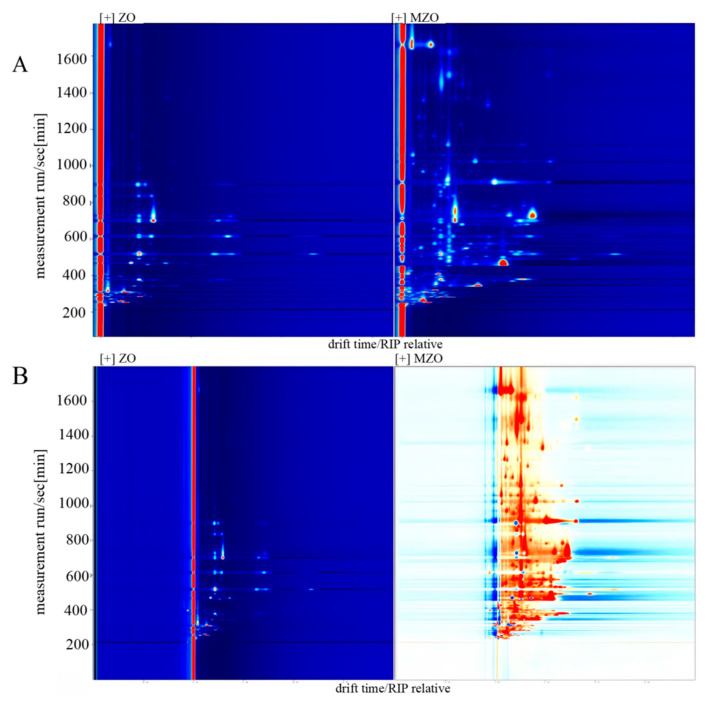
The information of VOCs determined using GC–IMS. (**A**) Topographical plots, (**B**) different comparison topographic plots.

The ion migration time and the position of the reactive ion peak (RIP) were normalized. The ordinate represents the retention time of the gas chromatography. The abscissa represents the ion migration time, and the vertical line at the abscissa 1.0 is the RIP peak. This revealed the total headspace compounds of the samples. Each point on the right of RI presented a volatile compound extracted from the samples. Color represented the signal intensity of the substance. White indicated lower intensity, and red indicated higher intensity [32]. Figure 6A shows that most of the signals appeared in the retention time of 200–800 s and the drift time of 1.0–1.8. Combining the above description and the two-dimensional top view of GC–IMS, the composition of volatile substances can be visually compared among different samples. Compared to ZO, MZO exhibited an increase in the number of flavor compounds as well as an elevation in their concentrations. The difference comparison model was applied to compare the aroma variety of samples (Figure 6B). The topographic plot of ZO was selected as a reference, and the topographic plot of MZO was deducted from the reference. If the volatile compounds were consistent, the background after deduction was white. However, red indicated that the concentration of the substance was higher than the reference, and blue indicated that the concentration of the substance was lower than the reference. Furthermore, compared to ZO, MZO exhibited a higher number of volatile components and a significant enhancement in odor intensity. These results suggest that microencapsulation has a certain effect on the preservation and enhancement of the flavor profile of *Zanthoxylum* oil. However, further research is needed to investigate the specific flavor-contributing substances by combining fingerprint analysis.

#### 2.4.2. Analysis of VOCs by GC–IMS

**Figure 7:**
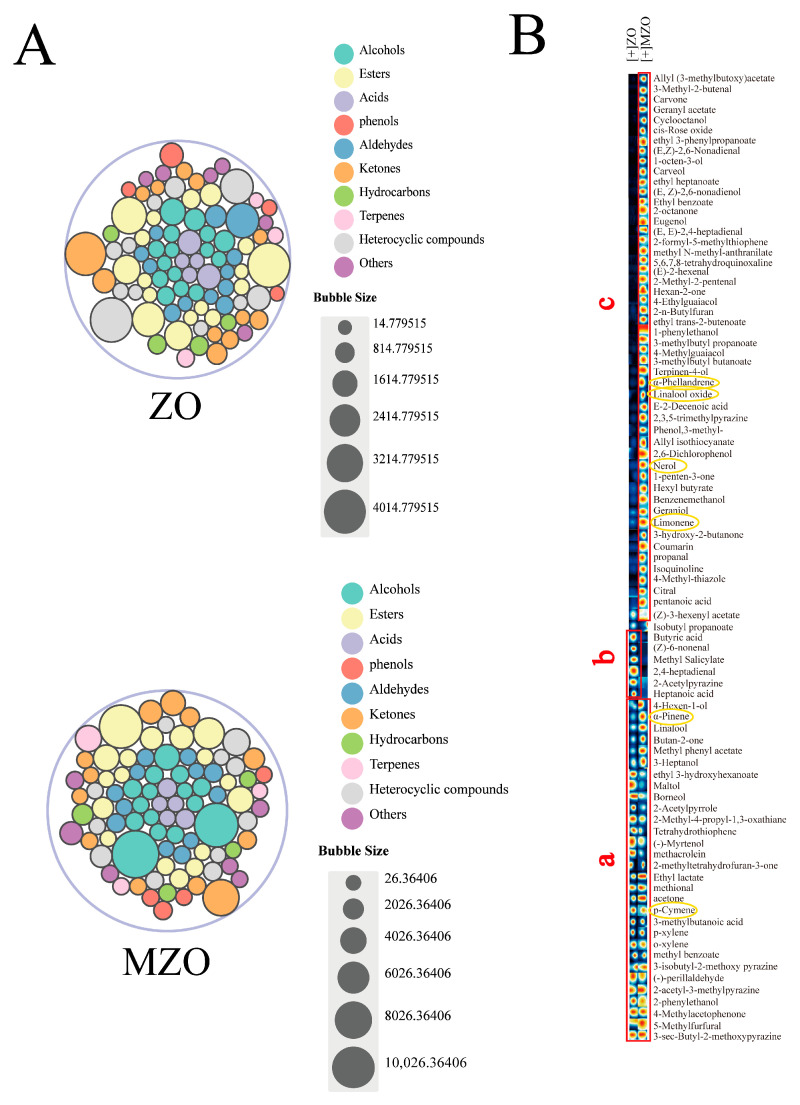
Investigation of GC–IMS. (**A**) Chart of the relative percentage content of samples based on the peak volume of VOCs, (**B**) fingerprint of VOCs.

In the two *Zanthoxylum* oil samples analyzed, a total of 90 VOCs were identified. As shown in Supplementary Table S2, these compounds include 18 esters, 15 alcohols, 5 acids, 4 phenols, 12 aldehydes, 12 ketones, 7 hydrocarbons, 11 heterocyclic compounds, and 6 other compounds. The samples were rich in common aromatic components such as limonene, α-pinene, ρ-cymene, phellandrene, nerol, and linalool oxide, which contribute to the intense woody, pine-like, and rose-like floral and sweet aromas of the *Zanthoxylum* oil [33]. Notably, certain volatile flavor compounds, including (E,Z)-2,6-nonadienol, 4-terpinenol, 4-hexen-1-ol, and borneol, have rarely been reported in previous studies on the volatile composition of *Zanthoxylum* oil [34]. As depicted in Figure 7A, esters (30.38%) and heterocyclic compounds (10.42%) were the predominant flavor components identified in ZO. Meanwhile, esters (29.08%) and alcohols (26.12%) were the predominant flavor components identified in MZO. Compared with ZO, MZO showed an increase in the content of alcohols (12.04–26.12%), an increase in the content of terpenes (1.39–3.53%), a decrease in the content of acids (5.77–2.72%), and a decrease in the content of aldehydes (10.29–4.62%).

To further differentiate the volatile flavor substances between ZO and MZO, all detected compounds in the GC–IMS spectra were selected to generate fingerprints using the Gallery Plot plug-in, as illustrated in Figure 7B. The changes in volatile compounds can be clearly observed through the fingerprint profiles. In the fingerprint profiles, the yellow circles highlight the main characteristic substances of *Zanthoxylum* oil. Compared to the other two regions, region c contains the highest proportion of these substances. Region a represents the organic compounds common to both *Zanthoxylum* oil samples, including 2-methoxy-3-sec-butylpyrazine, 5-methylfurfural, diethyl succinate, *p*-methyl acetophenone, phenyl ethanol, 2-acetyl-3-methylpyrazine, perillaldehyde, 2-methoxy-isobutylpyrazine, methyl benzoate, *o*-xylene, *p*-xylene, isovaleric acid, and *o*-cymene. Among these, phenyl ethanol, diethyl succinate, and 5-methylfurfural exhibit rose-like floral and pleasant aromas, which may contribute to the flavor quality of *Zanthoxylum* oil. Specifically, from regions b and c, it is evident that methyl 2-hydroxybenzoate, heptanoic acid, butanoic acid, (2*E*,4*E*)-hepta-2,4-dienal, and 1-pyrazin-2-ylethanone are the characteristic flavor substances in ZO, while the characteristic flavor substances in MZO were mainly alcohols and hydrocarbons, such as limonene, nerol, linalool oxide, phellandrene, carveol and 1-octen-3-ol. Meanwhile, the contents of aldehydes and ketones were also relatively high, including compounds such as neral, trans-2-hexenal, perillaldehyde, cyclooctanone, and carvone. The results indicate that, compared to the untreated *Zanthoxylum* oil, the microencapsulated *Zanthoxylum* oil exhibits better preservation of its VOCs, thereby retaining the original flavor profile of *Zanthoxylum* more effectively.

The results exhibit certain discrepancies compared to those obtained by GC–MS. This is primarily because GC–MS predominantly detects larger molecular weight and higher-concentration volatile compounds, whereas GC–IMS identifies mostly small molecular weight and low-concentration VOCs. Additionally, IMS demonstrates high sensitivity toward substances with functional groups possessing high electronegativity or proton affinity, such as amino, thiol, halogen groups, and organic compounds containing unsaturated bond structures like aldehydes, ketones, ethers, as well as aromatic compounds. Currently, most studies on the composition of *Zanthoxylum* oil rely on GC–MS for detection and analysis, with limited detection of compounds such as ketones, acids, and furans [35]. This is due to their small molecular weights and extremely low concentrations, making them difficult to detect [36]. However, this study demonstrates that GC–IMS can effectively compensate for the limitations of GC–MS, expanding the detection range of VOCs in samples and showing significant potential in the analysis of VOCs. The analytical results of GC–MS and GC–IMS exhibited certain discrepancies, primarily attributed to the fact that GC–MS predominantly detects higher molecular weight (C_9_–C_21_) and high-abundance volatile components, whereas GC–IMS identifies mainly lower molecular weight (C_2_–C_10_) and low-abundance volatile compounds. Thus, the combination of these two techniques compensates for their respective limitations, expands the detection scope of VOCs in samples, and provides a comprehensive reflection of variations in VOCs.

A notable limitation of this study arises from the absence of quantitative analysis using liquid chromatography (LC) for detecting amide compounds (specifically sanshool analogs) in the samples, constrained by current experimental conditions. To address this critical gap, future investigations will prioritize the optimization of experimental protocols through the incorporation of advanced LC instrumentation coupled with tandem mass spectrometry (LC–MS/MS), enabling precise characterization of the compositional profiles, quantitative dynamics, and structural transformations of key sanshool analogs in both native and microencapsulated *Zanthoxylum* oils. Such methodological refinements are anticipated to systematically elucidate the mechanistic interactions between microencapsulation matrices and bioactive constituents governing quality attributes, thereby establishing evidence-based guidelines for the industrial development of standardized *Zanthoxylum*-derived products with enhanced sensory and functional stability.

## 3. Materials and Methods

### 3.1. Materials and Reagents

The materials and reagents used in this study include *Zanthoxylum* oil, provided by Sichuan Yaomazi Food Co., Ltd. (Meishan, China), and *Zanthoxylum* oil, extracted by supercritical CO_2_ and supplied by Kunshan Sheng‘an Biotechnology Co., Ltd. (Kunshan, China). Additionally, Arabic gum (Shanghai Xintai Industrial Co., Ltd., Shanghai, China), sodium starch octenyl succinate (Guangzhou Licheng Industrial Co., Ltd., Guangzhou, China), mono- and diglycerides of fatty acids (Heilongjiang Weihengmei Trading Co., Ltd., Harbin, China), and sucrose esters of fatty acids (Henan Wanbang Chemical Technology Co., Ltd., Shangqiu, China) were all purchased from Jingdong Online supermarket. Throughout the experiment, distilled water was utilized as the experimental water source.

### 3.2. Microencapsulated Zanthoxylum Oil Production Process

The preparation process was adapted and optimized based on the method described by previous study [37,38]. Briefly, a specific amount of Arabic gum and sodium starch octenyl succinate was dissolved in distilled water at 60 °C, followed by constant-temperature stirring for 30 min. Subsequently, a measured quantity of *Zanthoxylum* oil was added and homogenized for 15 min. Mono- and diglycerides of fatty acids and sucrose esters of fatty acids were then incorporated, and the mixture was subjected to high-speed shear emulsification at 15,000 r/min for a designated duration to obtain the emulsion. Finally, the encapsulated *Zanthoxylum* oil powder was produced through spray drying and subsequent cooling.

### 3.3. Assessment of Physicochemical Characteristics

#### 3.3.1. Sensory Evaluation

Sensory evaluation was conducted using a semi-structured descriptive analysis method with a trained panel. Ten graduate students (5 males, 5 females; aged 23–25 years) from the Food Science program were recruited and participated voluntarily after completing a four-week training. The training focused on familiarization with key sensory attributes of *Zanthoxylum* oil, including aroma intensity and numbing sensation, with calibration using reference standards and practice with structured scoring forms.

The evaluation was designed to assess two primary descriptive attributes, including aroma and numbing sensation, and included an additional acceptability score as a supplementary indicator of perceived product quality. Attribute definitions and scoring criteria are summarized in Table 2. Panelists used structured interval scales to rate each attribute based on intensity and acceptability.

To minimize sensory fatigue, each sample was evaluated in triplicate, with a 15-minute rest interval between sessions. Samples (50 ± 0.5 g) were served in randomized, balanced order using odorless, disposable plates coded with random three-digit numbers. Evaluations were conducted in individual sensory booths under controlled conditions (temperature: 22 ± 1 °C; relative humidity: 50 ± 5%; illumination: 750 lux) to ensure reproducibility and eliminate external bias. The information and privacy of participants in the study were anonymized, and appropriate measures were taken to protect it. Consent was obtained from all participants before the open publication of the experimental data.

#### 3.3.2. Determination of Colorimetric Analysis

The chromaticity values of *Zanthoxylum* oil samples were measured at three positions near the center of each group using a colorimeter (NR200+, 3 nh Intelligent Technology Co., Ltd., Guangzhou City, China). Prior to measurements, the colorimeter was calibrated using a D65 whiteboard. Each measurement was conducted three times, and the average value was recorded. The color parameters assessed were L* (lightness), a* (red-green), b* (yellow-blue), C* (chroma), h* (hue angle), and Δ*E*. L* reflects brightness from black to white. Positive a* values indicate redness, and negative values indicate greenness. Positive b* values represent yellowness, and negative values represent blueness. Chroma (C*) describes the intensity or saturation of the color, and the hue angle (h*) specifies the dominant color tone. Together, these parameters enable comprehensive and quantitative evaluation of color characteristics in the samples. The equation of Δ*E* is presented as follows. *L*_0_, *a*_0_, and *b*_0_ are the parameters of control group (ZO).

$$\Delta E=\sqrt{(L^{*}-L_{0}{)}^{2}+(a^{*}-a_{0}{)}^{2}+(b^{*}-b_{0}{)}^{2}}$$

### 3.4. Analysis of Characteristic Flavor Compounds of Zanthoxylum Oil

#### 3.4.1. Analysis of E-Nose

For assessing overall odor characteristics, the Fox 4000 E-nose (Alpha MOS, Toulouse, France) was employed. This system includes 18 sensor chambers, a mass flow controller, and a microcontroller-based acquisition board. Sensor names follow Alpha MOS’s original coding convention (e.g., ‘LY’ in ‘LY2/LG’ represents the coating material type, ‘2’ indicates the version, and ‘LG’ denotes specific parameters), which are the officially recognized full names in the device’s technical documentation. Each sensor’s specific capabilities are outlined in Table 3. A 1.0 g homogeneous sample was placed into a 15 mL headspace vial, which was then sealed with double layers of plastic wrap. After equilibrating at room temperature for 30 min, the sample was subjected to E-nose detection and analysis using the headspace sampling method. The E-nose sampling parameters were set as follows: sample measurement interval of 1 s, automatic sensor cleaning time of 60 s, sensor zeroing time of 10 s, sample preparation time of 5 s, analysis sampling time of 70 s, and a sample flow rate of 400 mL/min. Each sample was analyzed five times, and stable values from the last three measurements were considered as the test results.

#### 3.4.2. Analysis of Volatile Compounds Using GC–MS

Volatile compounds were analyzed in *Zanthoxylum* oil using a GC–MS (SQ680 gas chromatography and mass spectrometer, PerkinElmer Inc., Waltham, MA, USA) system equipped with a polar DB-WAX capillary column (30 m × 0.25 mm × 0.25 μm; Agilent Technologies, Santa Clara, CA, USA). Sample preparation and injection conditions are as follows: samples, including 2.0 g of ZO and 2.97 g of MZO (ensuring consistent amounts of MZO in each sample), were placed into 20 mL dedicated headspace vials and sealed with PTFE and aluminum caps, with three vials prepared for each sample. The extraction and injection conditions were set as follows: incubation temperature at 60 °C for 30 min, injection needle temperature at 70 °C, transfer line temperature at 75 °C, dry purging for 120 s, desorption for 10 s, headspace vial pressurization/depressurization for 120 s, trap holding for 240 s, and trap cycling repeated 4 times. The qualitative and quantitative analysis of volatile compounds was performed by comparing mass spectra with those in the NIST mass spectral library (R match > 80%) and calculating retention indices (RIs) using a homologous series of n-alkanes (C_7_–C_40_) as references. Quantification was achieved by comparing the chromatographic peak areas of volatile compounds relative to that of 2-methyl-3-heptanone as the internal standard. All analyses were performed in triplicate.

The GC conditions are as follows: carrier gas (99.999% He) flow rate, 1.0 mL/min; injection port temperature, 250 °C; and initial temperature, 40 °C, then increased to 90 °C at 25 °C/min, held for 1 min, further increased to 105 °C at 1 °C/min, and finally raised to 200 °C at 3 °C/min, held for 2 min. The total analysis time was 51.67 min.

The MS conditions are ion source temperature 230 °C; electron ionization source; electron energy 70 eV; transmission line temperature 280 °C; electron multiplier voltage 1650 V; and mass scan range 45 (to avoid CO_2_ detection) to 450 *m*/*z*.

#### 3.4.3. Analysis of the Odor Activity Value

According to Guadagni’s aroma value theory, substances present in high concentrations but with very low thresholds in a sample are likely to be characteristic aroma compounds. The odor activity value (OAV) was calculated based on the ratio of each compound’s concentration to its perception threshold. Generally, when the OAV of a volatile substance is greater than 1, it is considered to contribute to the aroma profile of the sample. When the OAV exceeds 10, the compound is regarded as an important contributor to the aroma [40]. If the OAV is greater than 100, the compound is identified as a key aroma compound.

$$OAV=\frac{C}{T}$$

#### 3.4.4. Analysis of Volatile Compounds Using GC–IMS

Volatile compounds were analyzed in *Zanthoxylum* oil using GC–IMS (Flavor Spec, G.A.S., Dortmund, Germany). The headspace sampling are as follows: a 1.0 g sample was weighed and placed into a 20.0 mL headspace vial. The sample was incubated at 60 °C with an incubation speed of 500 r/min for 20 min. The injection needle temperature was set to 85 °C, and 500.0 μL of the sample was injected in spitless mode using nitrogen (purity: 99.999%) as the carrier gas. The cleaning time was set to 30 s.

The GC–IMS conditions are as follows: the analysis was performed using an MXT-5 chromatographic column (RESTEK, Bellefonte, PA, USA) with a length of 15 m, an internal diameter of 0.53 mm, and a film thickness of 1 μm. The column temperature was maintained at 60 °C, and the total analysis time was 25 min. High-purity nitrogen (purity ≥ 99.99%) was used as the drift gas. The flow rate was initially set at 2.0 mL/min, held for 2 min, then linearly increased to 10 mL/min over 10 min, further increased to 100 mL/min over 20 min, and finally increased to 150 mL/min over 25 min. The IMS temperature was set to 45 °C, and the desorption time was 25 min. After the analysis, the retention index (RI) of VOCs was determined using n-ketone C_4_–C_9_ as the reference. The RI and drift time of VOCs were matched with the NIST database and IMS database.

### 3.5. Statistical Analysis

All data were analyzed using IBM SPSS Statistics 26.0 (SPSS Inc., Chicago, IL, USA) and one-factor analysis of variance (ANOVA), and *p* < 0.05 was considered to be statistically significant. The characteristic fingerprint and the difference plots were generated using Gallery Plot (Version 1.2.6) and Reporter software (Version 1.4.03, G.A.S., Dortmund, Germany). Origin 2021 (Origin Lab Corporation, Northampton, MA, USA) was used for radar plot analysis, line chart analysis, and PCA analysis. OPLS-DA and VIP were performed using SIMCA software (Version 18.1, Umetrics, Umea, Sweden). Cluster heatmap was constructed using the TBtools-II software https://github.com/CJ-Chen/TBtools-II (accessed on 4 June 2025)

## 4. Conclusions

This study employed a multi-technique approach that integrated sensory evaluation, colorimetric analysis, E-nose, GC–MS, and GC–IMS to characterize the effects of microencapsulation on the volatile profile and sensory properties of *Zanthoxylum* oil. Sensory analysis revealed significant alterations in aromatic attributes between ZO and MZO, while numbing intensity and overall acceptability remained unchanged. Colorimetric and E-nose results confirmed distinct visual and olfactory differences, with MZO exhibiting reduced overall aroma intensity. GC–MS identified 43 VOCs, with terpenes as the dominant class (69.7% in ZO, 68.2% in MZO), and eight key aroma components were pinpointed using OAV and VIP analysis. GC–IMS detected 90 VOCs, revealing compositional shifts. Esters and heterocyclic compounds predominated in ZO, whereas esters and alcohols dominated in MZO, with MZO showing increased alcohol (12.04–26.12%) and terpene (1.39–3.53%) contents but reduced acid (5.77–2.72%) and aldehyde (10.29–4.62%) levels. These findings highlight that microencapsulation modifies volatile profiles while preserving critical sensory attributes like numbing sensation, providing a scientific basis for optimizing microencapsulation processes to maintain flavor quality and facilitate *Zanthoxylum* oil resource utilization. The integrated analytical framework demonstrated enhanced capability in deciphering complex volatile transformations, underscoring its utility for comprehensive food quality evaluation.

## Figures and Tables

**Table 1:** Color difference analysis.

Sample	L*	a*	b*	C*	h*	Δ*E*
ZO	67.28 ± 0.20 ^b^	4.73 ± 0.06 ^b^	19.71 ± 0.29 ^b^	20.27 ± 0.27 ^b^	76.01 ± 0.33 ^a^	20.12 ± 0.10 ^a^
MZO	88.07 ± 0.11 ^a^	5.74 ± 0.05 ^a^	21.93 ± 0.26 ^a^	22.66 ± 0.26 ^a^	75.33 ± 0.05 ^a^	10.13 ± 0.23 ^b^

Notes: The experimental results are presented as mean ± standard deviation of triplicate analysis. The different letters show a significant difference between samples according to Duncan’s multiple range test (*p* < 0.05).

**Table 2:** Sensory attributes and scoring criteria used in trained panel evaluation of *Zanthoxylum* oil samples.

Sensory Attribute	Descriptive Criteria	Score
Aroma Intensity	1–10: Absent or weak aroma	1–30
	11–20: Moderate aroma without off-notes	
	21–30: Strong, characteristic Zanthoxylum aroma	
Numbing Sensation	1–13: No or very slight numbness	1–40
	14–27: Noticeable and lingering numbness	
	28–40: Strong trigeminal numbing effect	
Acceptability	1–10: Unacceptable	1–30
	11–20: Moderately acceptable	
	21–30: Highly acceptable	

Notes: Acceptability was included to provide additional context for sensory perception but was not treated as a descriptive attribute in the formal analysis.

**Table 3:** Performance description of the E-nose sensors. Reprinted from Ref. [39].

Sensors	Performance Description	Sensors	Performance Description
LY2/LG	Sensitive to oxidizing gas	P40/1	Sensitive to fluorine
LY2/G	Sensitive to ammonia, carbon monoxide	T70/2	Sensitive to aromatic compounds
LY2/AA	Sensitive to ethanol	PA/2	Sensitive to ethanol, ammonia/organic amines
LY2/Gh	Sensitive to ammonia/organic amines	P30/1	Sensitive to polar compounds (ethanol)
LY2/gCT1	Sensitive to hydrogen sulfide	P40/2	Sensitive to heteroatom/chloride/aldehydes
LY2/gCT	Sensitive to propane/butane	P30/2	Sensitive to alcohol
T30/1	Sensitive to organic solvents	T40/2	Sensitive to aldehydes
P10/1	Sensitive to hydrocarbons	T40/1	Sensitive to chlorinated compounds
P10/2	Sensitive to methane	TA/2	Sensitive to air quality

## Data Availability

The original contributions presented in this study are included in the article/Supplementary Material. Further inquiries can be directed to the corresponding authors.

## Supplementary Materials

The following supporting information can be downloaded at: https://www.mdpi.com/article/10.3390/molecules30163366/s1, Table S1: GC-MS analysis of VOCs; Table S2: VOCs identified by GC-IMS.
