# Supplementary material for: The Effects of Microencapsulation Technology on the Flavor Quality of Zanthoxylum Oil Based on E-Nose, GC–IMS, and GC–MS
Source: Molecules. 2025 Aug 13;30(16):3366. doi: 10.3390/molecules30163366 (PMC12388017; doi:10.3390/molecules30163366)
Supplement: Supplementary file 1 [file molecules-30-03366-s001.zip › molecules-3720137-supplementary.pdf]

Table S1. GC-MS analysis of VOCs.

No	Volatile compound	CAS	R Match	RI[a]	Relative content of components (Peak area, %)		Threshold (mg/kg)	OAV		Odor description
					CK	MZO		CK	MZO	
					Hydrocarbons					
1	2,6-Dimethyl-2-trans-6-octadiene	2609-23-6	834	1060	ND	0.04±0.00	ND	ND	ND	ND
2	(3E)-penta-1,3-diene	504-60-9	892	995	ND	0.01±0.00	ND	ND	ND	ND
3	2-methylbut-2-ene	513-35-9	841	1050	ND	10.33±0.18	ND	ND	ND	ND
4	(3E,5E)-octa-1,3,5-triene	33580-04-0	820	1425	ND	0.07±0.00	ND	ND	ND	ND
5	1,3,5-Cyclooctatriene	1871-52-9	819	1030	ND	0.34±0.00	ND	ND	ND	ND
6	1,4-Pentadiene	591-93-5	878	600	0.01±0.00	0.35±0.00	ND	ND	ND	ND
7	eucalyptol	470-82-6	910	1211	3.09±0.05	2.35±0.05	0.0011	2807.58±58.05	2133.03±50.33	minty,herbaceous, woody
8	o-Cymene	527-84-4	825	1268	0.80±0.01	0.01±0.00	0.005	159.93±2.05	2.0±0	spicy, bitter
					Terpenes					
9	β-Terpinene	99-84-3	901	1206	0.09±0.00	0.06±0.00	ND	ND	ND	ND
10	β-Phellandrene	555-10-2	895	1195	0.46±0.01	0.04±0.00	0.04	12.81±0.22	1.11±0	woody, herbal, green
11	α-Phellandrene	99-83-2	879	1158	3.32±0.07	3.32±0.07	0.09	17.06±0.30	15.25±0.34	herbceous, woody, floral
12	α-Pinene	80-56-8	913	1015	0.65±0.01	0.64±0.01	0.04	15.89±0.18	15.64±0.41	pine, earthy, resinous
13	(+)-Limonene	5989-27-5	829	1035	ND	0.01±0.00	0.03	ND	0.29±0	citrusy, sweet, fruity
14	gamma-Terpinene	99-85-4	834	1223	4.67±0.07	3.76±0.06	1.00	4.66±0.09	3.76±0.08	woody, citrus, herbal
15	Myrcene	123-35-3	865	1160	10.35±0.21	8.32±0.13	0.0012	8623.05±216.83	6936.39±131.69	green, spicy
16	Terpinolene	586-62-9	875	1282	1.33±0.02	0.33±0.01	0.20	6.64±0.12	1.66±0.04	pine, floral
17	Limonene	138-86-3	862	1185	29.7±0.37	26.63±0.54	0.20	148.50±2.24	133.16±3.28	sweet, citrus
18	Ocimene	13877-91-3	837	1025	2.73±0.06	2.32±0.04	ND	ND	ND	ND
19	1,3,8-p-Menthatriene	18368-95-1	864	1390	2.93±0.05	2.75±0.04	0.02	195.11±4.29	183.33±3.34	minty, herbal, camphoraceous
20	α-Terpinene	99-86-5	826	1195	1.45±0.02	1.30±0.02	0.09	17.06±0.30	15.25±0.34	herbaceous, woody, floral
21	β-Pinene	127-91-3	901	1100	6.49±0.12	4.28±0.05	0.14	46.39±1.09	30.56±0.44	resinous, spicy, green
22	Camphene	79-92-5	908	1057	0.10±0.00	ND	30.00	0±0	ND	earthy, herbal, camphoraceous
23	3-Carene	13466-78-9	829	1410	1.36±0.01	0.95±0.02	0.77	1.76±0.02	1.23±0.03	balsamic, spicy, piney
24	1-methyl-5-prop-1-en-2-ylcyclohexene	1461-27-4	808	1460	ND	0.02±0.00	ND	ND	ND	ND

25	p-Mentha-2,4(8)-diene	586-63-0	805	1270	0.03±0.00	0.01±0.00	ND	ND	ND	ND
					Alcohols					
26	pentan-1-ol	71-41-0	956	1255	ND	0.03±0.00	0.15	ND	0.20±0	fruity, grassy
27	(3E,5E)-nona-3,5-dien-7-yn-2-ol	43142-43-4	871	1285	ND	0.01±0.00	ND	ND	ND	ND
28	Methanethiol	74-93-1	840	675	0.01±0.00	ND	ND	ND	ND	sulfur, gasoline, garlic
29	Linalool	78-70-6	890	1553	6.17±0.08	1.44±0.03	0.00022	28063.63±432.46	6563.64±168.36	flowery, citrusy
30	Linalool oxide	5989-33-3	847	1423	3.50±0.06	1.28±0.03	0.10	35.01±0.72	12.77±0.33	floral, herbaceous, woody, citrus
31	2,6-dimethyl-2,7-Octadiene-1,6-diol	103619-06-3	831	1450	0.05±0.00	ND	ND	ND	ND	ND
					Aldehydes					
32	3-hydroxy-2,2-dimethyl propanal	597-31-9	808	1060	ND	1.71±0.03	ND	ND	ND	ND
33	pentanal	110-62-3	854	984	ND	0.04±0.00	0.01	ND	3.33±0	green, fruity, nutty
34	hexanal	66-25-1	942	1097	ND	0.02±0.00	0.005	ND	4.00±0	green, herbaceous, fresh
35	hept-4-enal	929-22-6	961	1080	0.04±0.00	ND	0.01	4.00±0	ND	creamy, potato-like
36	octanal	124-13-0	958	1295	4.63±0.1	4.87±0.07	0.00058	7885.29±202.98	8288.47±144.61	citrusy, sweet, waxy
37	butanedial	638-37-9	876	1150	ND	0.02±0.00	ND	ND	ND	ND
					Esters					
38	pentyl propanoate	624-54-4	864	1350	0.58±0.01	0.01±0.00	0.085	6.83±0.12	0.12±0	buttery, rum-like, floral
39	3-methylbutyl acetate	123-92-2	873	1180	ND	0.01±0.00	0.067	ND	66.67±0	fruity, ethereal
					Ketones					
40	2,3,5-trimethyl-4-methylidenecyclopent-2-en-1-one	29765-85-3	809	1400	1.71±0.03	1.24±0.02	ND	ND	ND	ND
41	4-methylhexan-2-one	105-42-0	820	850	ND	0.01±0.00	0.0008	ND	12.35±0	camphor, pungent, woody
42	4,6-dimethylheptan-2-one	19549-80-5	811	1100	ND	0.07±0.00	ND	ND	ND	ND
43	p-Mentha-2,8-diene	5113-87-1	824	1160	ND	0.01±0.00	ND	ND	ND	ND

Notes:[a]:Retention indices were determined using a homologous series of n-alkanes.

Table S2. VOCs identified by GC-IMS.

Component Name	structural formula	RI	Rt [sec]	Odor description
	Alcohols			
oct-1-en-3-ol	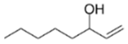	1433.6	1665.507	ND
Nerol	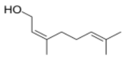	1203.7	859.945	fresh roses
Myrtenol	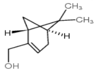	1197.9	836.656	grass
geraniol	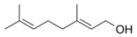	1236.5	989.276	fresh roses
Carveol	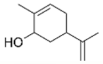	1184.7	784.627	ND
(2E,6Z)-nona-2,6-dien-1-ol	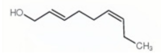	1151.2	658.885	fresh cucumber-like aroma
Linalool oxide	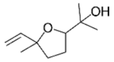	1069.4	454.818	ND
cyclooctanol	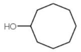	1171.2	731.346	ND
hex-4-en-1-ol	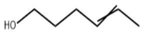	910.0	302.160	green, leafy aroma
4-terpineol	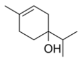	1169.5	724.518	woody and floral aroma
2-phenylethanol	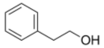	1085.7	494.947	rosy and floral
Linalool	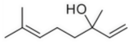	1084.8	492.889	floral with a touch of citrus
phenyl methanol	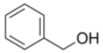	1025.0	397.054	sweet, floral aroma
heptan-3-ol	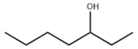	912.4	303.481	earthy, nutty, roasted, cocoa-like

borneol	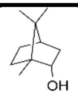	1165.6	709.163	camphoraceous aroma
		Esters		
methyl 2-(methylamino)benzoate	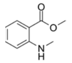	1385.7	1501.910	grape-like aroma
Geranyl acetate	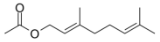	1373.5	1460.445	floral, fruity aroma
ethyl 3-phenylpropanoate	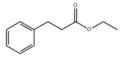	1369.8	1447.629	fruity, floral aroma
Allyl amyl glycolate	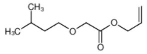	1247.3	1028.917	ND
diethyl butanedioate	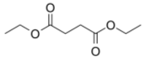	1198.0	837.151	fruity and wine-like
3-methylbutyl propanoate	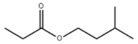	1188.7	800.483	fruity and banana-like
ethyl heptanoate	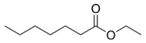	1108.4	551.824	fruity with a wine-like nuance
methyl benzoate	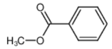	1098.5	526.981	fruity, sweet, and floral
3-methylbutyl butanoate	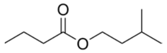	1073.1	463.690	fruity banana-like
methyl 2-phenylacetate	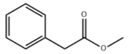	1163.5	700.861	honey-like
Ethyl lactate	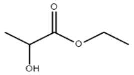	796.6	254.049	fruity and creamy
3-Hexenylacetate	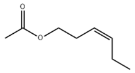	984.4	344.244	fresh green apple
2-methylpropyl propanoate	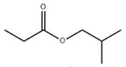	876.0	286.448	fruity and rum-like
methyl 2-hydroxybenzoate	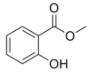	1170.4	728.201	wintergreen-like aroma
3-isothiocyanatoprop-1-ene	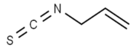	839.3	271.464	ND

ethyl benzoate	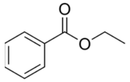	1156.2	671.898	sweet, fruity, and balsamic
ethyl 3-hydroxyhexanoate	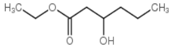	1134.2	616.297	fruity and wine-like
hexyl butanoate	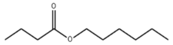	1133.0	613.340	fruity, pineapple-like aroma
	Acids			
2-Decenoic acid	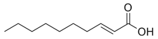	1336.3	1333.035	ND
3-methylbutanoic acid	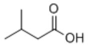	873.8	285.563	pungent cheesy aroma
heptanoic acid	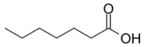	1075.5	469.493	pungent, rancid odor
butanoic acid	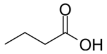	821.5	264.187	pungent, cheesy, and sour flavor
pentanoic acid	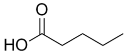	924.6	310.307	ND
	Phenols			
eugenol	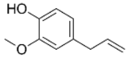	1348.2	1373.746	warm, spicy aroma
4-ethylguaiacol	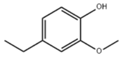	1256.7	1061.126	smoky, spicy, and clove-like flavor
3-hydroxy-2-methylpyran-4-one	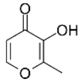	1213.0	896.614	sweet, caramel-like, fruity
2-methoxy-4-methylphenol	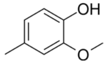	1184.6	784.131	smoky, spicy, and clove-like
	Aldehydes			
3-methylbut-2-enal	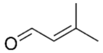	1231.6	969.950	ND
Menthol	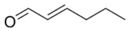	1227.4	953.598	fresh, green, grassy, leafy
Methyl benzoate	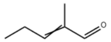	1183.8	781.158	ND
Neral	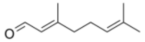	1242.6	1013.060	lemon-like flavor
(2E,6Z)-nona-2,6-dienal	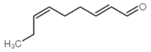	1118.1	576.076	fresh, green, cucumber-like

5-methylthiophene-2-carbaldehyde	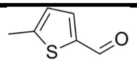	1103.0	538.219	ND
2-methylprop-2-enal	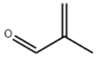	875.2	286.106	ND
3-methylsulfanylpropanal	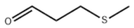	914.3	304.580	pungent, savory, and meaty aroma
6-Nonenal	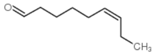	1075.8	470.356	ND
propanal	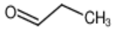	778.2	246.532	pungent, sharp, and fruity aroma
Perillyl aldehyde	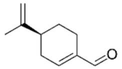	1134.1	615.931	ND
2,4-Heptadienal	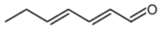	984.8	344.821	nutty, fatty, and fried aroma
	Ketones			
octan-2-one	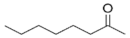	1304.9	1225.981	fruity, citrus-like aroma
3-hydroxybutan-2-one	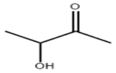	1257.1	1062.612	creamy, buttery, and slightly sweet aroma
α-carvone	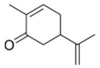	1246.2	1025.448	refreshing, minty-sweet, herbal aroma
cyclopentanone	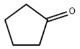	1159.3	684.320	pungent, sweet-minty
hexan-2-one	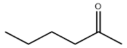	1118.1	576.076	ND
butan-2-one	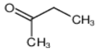	896.5	294.799	ND
propan-2-one	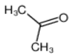	815.9	261.900	ND
2-oxopropyl acetate	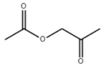	808.5	258.885	ND
thiolane	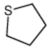	801.1	255.884	sulfurous, onion-like, and meaty aroma
2-methyloxolan-3-one	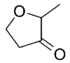	790.1	251.378	mild roasted aroma

1-(4-methyl phenyl) ethanone	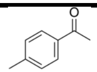	1136.7	622.590	floral, cherry, honey, woody
thiolan-3-one	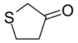	944.2	321.343	onion-like, roasted and meaty aroma
hydrocarbons				
undecane	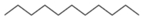	1108.6	552.416	ND
1,4-xylene	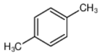	841.2	272.251	ND
1,2-xylene	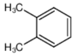	898.4	295.615	pungent, aromatic, sweet, and solvent-like
p-cymene	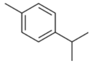	1009.5	376.844	citrusy, herbal, and slightly woody aroma
terpenes				
α-Pinene	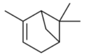	914.8	304.851	fresh, pine-like, woody aroma
α-Phellandrene	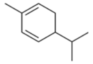	1011.3	379.289	ND
Limonene	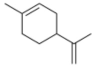	1004.0	369.761	citrus, piney, fresh, woody, slightly sweet
Heterocyclic compounds				
2,5-dimethylpyrazine	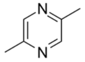	1345.1	1363.191	earthy, nutty, roasted, cocoa-like, musty
2-methyl-4-propyl-1,3-oxathiane	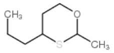	1134.4	616.889	ND
1-(1H-pyrrol-2-yl) ethanone	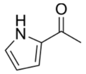	1024.7	396.676	nutty, licorice, toasted, popcorn-like aroma
2,3,5-trimethylpyrazine	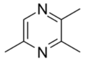	1004.4	370.324	ND
2-butylfuran	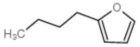	896.8	294.909	ND
2-methoxy-3-(2-methylpropyl) pyrazine	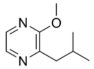	1166.1	711.162	ND

2-butan-2-yl-3-methoxypyrazine	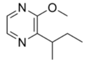	1095.0	518.198	green, earthy, bell pepper, and potato-like aroma
1-(3-methylpyrazin-2-yl) ethanone	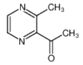	1093.6	514.742	ND
1-pyrazin-2-ylethanone	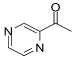	1011.9	379.999	ND
4-methyl-1,3-thiazole	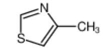	767.8	242.292	ND
5-methylfuran-2-carbaldehyde	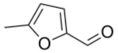	970.6	336.169	ND
		Others		
isoquinoline	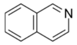	1286.6	1163.406	anise-like, and bitter almond
5,6,7,8-tetrahydroquinoxaline	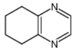	1272.5	1115.137	nutty, roasted, and cereal-like flavor
1-Phenyl-2-aminoethanol	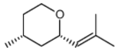	1108.6	552.416	ND
1-(propyldisulfanyl)propane	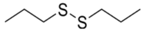	1089.9	505.687	onion and garlic aroma
1-propylsulfanylpropane	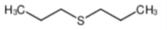	874.4	285.785	sulfurous, pungent
3-prop-2-enylsulfanylprop-1-ene	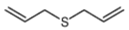	867.5	282.961	garlicky, pungent, sulfuric, onion-like

Notes: Odor description is taken from the Chemical Book database. RI = retention index, Rt = retention time, ND = no data.
